# Transcriptomic Variation during Spermiogenesis in Mouse Germ Cells

**DOI:** 10.1371/journal.pone.0164874

**Published:** 2016-11-11

**Authors:** Haiyang Zuo, Junfang Zhang, Liuguang Zhang, Xiaoxia Ren, Xiaoli Chen, Haisheng Hao, Xueming Zhao, Dong Wang

**Affiliations:** 1 The Key Laboratory for Farm Animal Genetic and Utilization of Ministry of Agriculture of China, Institute of Animal Science, Chinese Academy of Agricultural Science, Beijing, 100193, China; 2 JiLin Agriculture University Animal Science and Technology, Changchun, 130118, China; 3 HaiKou Mary Hospital Reproductive Medicine Center, Haikou, 570100, China; Centre de Recherche en Cancerologie de Lyon, FRANCE

## Abstract

To explore variations in the transcription activity during spermiogenesis, round and elongated spermatids were collected from ICR/CD1 model mice using laser capture microdissection (LCM) and cauda epididymal sperm samples. The transcripts were sequenced using RNA-seq, and the reads were mapped to mm9. The majority of the reads (70%) in the round and elongated spermatids were mappable to known and predicted exons, but that in sperm was only 9%. The results of the distribution of reads suggested that alternative splicing was more complicated in sperm than in round and elongated spermatids. In the 19,127 genes, we detected the expression of 5,104 de novo genes and 91,112 alternative splicing events, and 12,105 were differentially expressed. Gene ontology (GO), InterPro domains, and KEGG revealed changes in gene transcription, mitochondrial protein translation, cellular components, and energy metabolism during spermiogenesis. The results provided considerable information about alternative splicing events, differentiallly expressed genes (DEGs), and novel transcriptions during spermiogenesis in mice.

## Introduction

Round spermatids undergo metamorphose into spermatozoa. During this process, most of the germ cell cytoplasm is lost, the histones are replaced by transition proteins, and these proteins are eventually transformed into protamines [1,2]. Spermatid chromatin is tightly packaged [1] and the transcription activity of spermatid is gradually lost [3]. In recent years, many types of transcripts have been detected in sperm, including those needed to repackage chromatin, and small RNAs. According to recent research, histones in the nucleus are not entirely replaced. Approximately 10% of the sites remain histones in the nucleosome in mouse spermatozoa [4,5], which makes the chromosome of these sites a slack spatial structure and may result in some transcriptionally active regions.

Sperm transcripts remain stable as ribonucleoprotein particles [6] and ultimately contribute to the zygote transcriptome and proteome (in the case of translation of introduced mRNAs), and therefore play functional roles in the zygote or in early embryogenesis [6]. As the mRNA population specifies the character of the cell line and helps govern its present and future activities, transcriptome analysis is now used as a phenotyping approach. The transcriptome of haploid germ cells contains genes with special complicated structure, which poses certain challenges [7,8]. Detailed study on the characteristics of transcription in spermiogenesis can help to understand the metamorphosis and genetic nature of spermiogenesis, and can be applied to develop novel clinical diagnostic tests of the sperm quality at a molecular level. Currently, sperm quality evaluation is limited to parameters such as the sperm count, morphology and motility. This new molecular approach may become a key for developing male contraceptive pills and infertility treatment. Thus far, no studies have explored the dynamic transcriptomic variations of haploid germ cells from the round spermatid stage to the spermatozoa.

To explore the expression variation at the transcriptomic level and the transcription activity at nucleotide resolution in spermiogenesis, we obtained haploid germ cells at three stages of spermiogenesis in an ICR mouse model: round spermatids, elongated spermatids, and cauda epididymal sperms, which were enriched and sequenced for the first time. The data from this study can be used to complement what is currently known about the composition of sperm at transcriptome level and their origin. Thus, this study reveals that the development of germ cells during spermiogenesis results from specific transcription pattern of the genome; Our findings can also help expand the scope of targeted molecular studies related to fertilization.

## Materials and Methods

### Cell sample collection

Mice were housed at 20–25°C under natural lighting, with food and water provided ad libitum. All animal care and sample collection procedures were approved and conducted in accordance with the Experimental Animals Standard Assembly (ISBN 9787506664486) of Institute of Laboratory Animal Science, Chinese Academy of Medical Sciences (CAMS), and all procedures used in the present study were approved by CAMS. Twenty healthy fertile white male ICR/CD1 mice (Vital River Laboratory Animal Technology Co. Ltd, Beijing China) of 8- to- 9-week-old were randomly chosen to use in this study. For spermatid collection, one testis of each mouse was used for round spermatids, and the other one for elongated spermatids. two sample pools of round (R1 & R2) and elongated (L1 & L2) spermatids were constructed from each testis. Also, two sperm pools (M1 & M2) were constructed by collecting sperms from all the epididymides.

Mice were sacrificed by decapitation, and the testes were dissected and snap-frozen in liquid nitrogen for subsequent collection of round and elongated spermatids. Sperm samples were harvested after swim-up from the cauda epididymis. Two biological replicates were performed for each sample. After the frozen testis was solidified with optimal cutting compound (OCT), 5-μm-thick cryosections were obtained using a Leica CM1950 (Leica Microsystems; Wetzlar, Germany), and the sections were mounted towards the center of a room-temperature microslide (RNAse-free polyethylene naphthalate [PEN]-coated slides, Leica, Germany). The slides were immediately placed in a microslide box in dry ice and stained and dehydrated using the HistoGene LCM frozen section staining kit. The cryosections were laser microdissected using a Leica LMD7000 (Leica, Germany) according to the manufacturer’s protocol. The UV laser microbeam was coupled with the epifluorescence illumination port of the microscope. Based on the microscopic images, the optimal settings of microdissection parameters are as followings: power, 15; aperture, 1; speed, 6; specimen balance, 20; head current, 77%; pulse frequency, 120. Round or elongated spermatids were screened on the basis of their morphological characteristics. Microdissection was performed by cutting along the cell edge with a focused laser beam. The target cell was separated and dropped into a microfuge cap moistened with 35 μL lysis buffer. To minimize the risk of RNA degradation, cell collection should be completed within 30 min for each cryosection. In our study, 500–1,000 cells were collected for per cryosection according to the cryosection’s condition.To avoid RNA degradation, harvested cell samples were soaked in lysis buffer and kept in liquid nitrogen till enough cell samples were collected. R1 and R2 samples contained 2.5–2.7 × 10^4^ and 2.7–3.0 × 10^4^ round spermatids, respectively, and L1 and L2 samples contained 2.9–3.1 × 10^4^ and 3.0–3.2 × 10^4^ elongated spermatids, respectively, and M1 and M2 samples contained 2.9 × 10^7^ and 3.2 × 10^7^ cauda epididymis sperms, respectively.

### RNA isolation and amplification

Total RNA was extracted using an Arcturus PicoPure RNA isolation kit (Life Technology, Carlsbad, USA) according to the manufacturer’s instructions. One round of linear amplification was performed to amplify the poly(A) RNA fractions of total RNA using a MessageAmp^™^ II aRNA amplification kit (Life Technology, Carlsbad, USA) according to the manufacturer’s instructions. At least 450 ng of total RNA was used for the amplification of each replicate. RNA purity was checked using a spectrophotometer (NanoPhotometer^®^; Implen, CA, USA). RNA concentration was measured using Qubit^®^ RNA Assay Kit in a Qubit^®^ 2.0 Flurometer (Life Technology, CA, USA). RNA integrity was assessed using the RNA Nano 6000 Pico Assay Kit in a Bioanalyzer 2100 system (Agilent Technology, CA, USA).

### Library preparation and Illumina sequence analysis

According to the manufacturer’s recommendations, sequencing libraries were generated using TruSeq^™^ RNA sample preparation kit (Illumina, San Diego, USA). 200 ng of mRNA per sample was used as the input material for each library, and six index codes were added to the attribute sequences of each sample. Fragmentation was carried out using divalent cations under elevated temperature in Illumina proprietary fragmentation buffer. First-strand cDNA was synthesized using random oligonucleotides and SuperScript II Reverse Transcriptase. Second-strand cDNA synthesis was subsequently performed using DNA polymerase I and RNase H. The remaining overhangs were converted into blunt ends via exonuclease/polymerase activities. After adenylation of the 3′-ends of DNA fragments, Illumina PE adapter oligonucleotides were ligated to prepare for hybridization. In order to preferentially select 200-bp cDNA fragments, the library fragments were purified with an AMPure XP system (Beckman Coulter, Beverly, USA). DNA fragments with ligated adaptor molecules on both ends were selectively enriched using Illumina PCR Primer Cocktail in a 10-cycle PCR reaction. The products were then quantified using Bio-Rad CFX96 fluorescent quantitative PCR (Bio-Rad, USA) and Bio-Rad IQ SYBR green kit (Bio-Rad, USA).

The index-coded samples were clustered on a cBot Cluster Generation System using TruSeq PE Cluster Kit v3-cBot-HS (Illumina, San Diego, USA) according to the manufacturer’s instructions. After cluster generation, the libraries were sequenced on an Illumina Hiseq 2000 platform, and 100-bp paired-end reads were generated.

Raw data (raw reads) from FASTQ were first processed through Novogene Perl scripts. Clean reads were obtained by removing the adapter sequence from the reads, reads containing ploy-N, and low-quality reads from the raw data. At the same time, Q20, Q30, GC content, and sequence duplication level of the clean data were calculated. All the downstream analyses were performed on high-quality clean data.

### Mapping of reads to the mouse reference genome

The mouse reference genome (mm9) and gene model annotation files (mouse NCBIM37.65) were downloaded from the mouse genome website (ftp://ftp.ensembl.org/pub/release-69/fasta/mus_musculus/dna/). An index of the reference genome was built using Bowtie (version 0.12.8). Paired-end clean reads were aligned to the reference genome using TopHat v1.4.0, allowing for up to three mismatches since using a smaller number of allowed maximum mismatches did not affect the analysis. Only the reads that mapped uniquely were included in the analysis, unless indicated otherwise. TopHat can generate a database of splice junctions based on the gene model annotation files, and thus can better interprete mapping results than other non-splice mapping tools.

### Quantification of gene expression and analysis of differential expression

The number of reads mapped to each gene was counted using HTSeq (version 0.5.3). The reads per kilobases per millionreads (RPKM) of each gene was calculated based on the gene length and read counts mapped to—it. Since RPKM simultaneously considers the effect of sequencing depth and gene length for the read count, it is currently the most commonly used method to estimate gene expression levels [9].

Differential expression analysis of three groups of samples (two biological replicates per group) was performed using the DESeq R package (version 1.8.3) according to a previous study [4]. The P values were adjusted using the Benjamini & Hochberg method [10]. The corrected P value of 0.05 was set as the threshold for significant differential expression.

### Functional analysis of differentially expressed genes (DEGs)

Gene ontology (GO) enrichment analysis of DEGs was implemented by the GOseq R package, in which gene length bias was corrected. The resulting GO terms from the “biological process” taxonomy with corrected P value of less than 0.05 were considered significantly enriched in DEGs.

DAVID [5] was used for gene enrichment analysis, including gene ontology (GO) terms, InterPro domains, and KEGG pathways [11].

### Prediction of novel transcripts and alternative splicing analysis

To construct and identify both known and novel transcripts from TopHat alignment results, Cufflinks (version 1.3.0) was used to perform Reference Annotation Based Transcript (RABT) assembly. Astalavista (version 2.2) was used to estimate the alternative splicing events both in and among the groups based on the results of the Cuffmerge and Cuffcompare modules in the Cufflinks package.

### Validation of RNA-seq by real-time PCR

To validate the differential gene expression levels, ten from 12,105 DEGs were randomly selected for quantitative real-time PCR (qPCR) analysis. Cell sample collection and total RNA extraction were carried out as described for RNA seq experiment. All primers (Table D in S2 File) were designed using Primer 5, and spanned an intron each. β-Actin was used as the housekeeping gene to normalize the qPCR expression level [12,13]. The total reaction volume was 15 μL, and contained 7.5 μL of 2× Sybr Green qPCR mix, 0.5 μL of forward primer (10 μM), 0.5 μL of reverse primer (10 μM), 1 μL of template cDNA, and 5.5 μL of nuclease-free water. qPCR was performed on an ABI StepOnePlus 7500 Sequence Detection System using the following parameters: initial denaturation at 95°C for 10 min; denaturation at 95°C for 20 s and annealing at 60°C for 30 s for 40 cycles; dissociation at 95°C for 15 s, 60°C for 30 s, and 9°C for 15 s. The comparative CT method (2^–ΔΔCT^ method) was used to analyze the expression level of genes.

## Results

### The transcriptome differences among spermatids and sperm

To characterize the dynamic expression variation at the transcriptional level during spermiogenesis in mouse, we generated approximately 73, 63, and 32 million (200)-bp reads mapped to unique sites in the mouse genome from round spermatids, elongated spermatids, and sperm samples, respectively. More than 67% of reads mapped uniquely to the genome, allowing up to 3 mismatches; among these uniq-mapped reads, 19.2%, 19.2%, and 0.5% of the reads were mapped to unique splice junctions of round spermatids, elongated spermatids, and sperm samples, respectively. All the primary sequence read data for both replicates of the three types of germ line cells are provided in Table A in S2 File.

**Fig 1:**
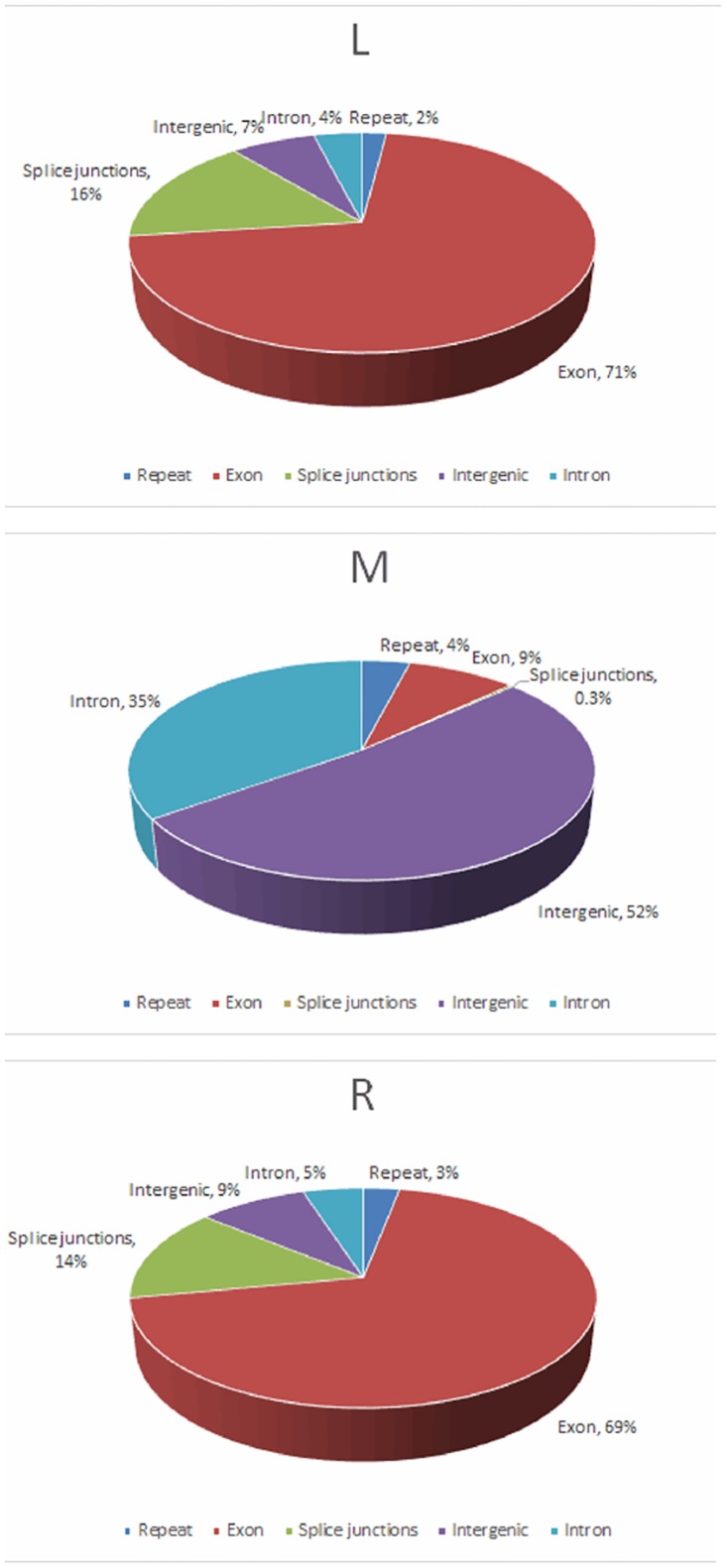
Distribution of mapped reads to the mouse annotated genome. R, round spermatid sample; L, elongated spermatid sample; M, sperm sample.

Summing the reads over an entire transcriptome (Fig 1), the genic distribution of reads in each round and elongate spermatid sample showed that majority (69–71%) reads mapped to known and predicted exons and only 4–5% mapped to introns; 7–9%, to intergenic regions; and 14–16%, to splice junctions. On the contrary, the distribution of reads in the sperm was very different: approximately 35% of the reads mapped within introns, an unexpectedly high percentage of 52% mapped to intergenics, and only 9% of the reads fell within the exons.

### The differences of alternative splicing events among spermatids and cauda epididymal sperm

Five types of common distinct splices, namely, intron retention (IR), exon skipping (SKIP), alternative 5′- or 3′- exon splicing site (AE), alternative first exon (transcription start site, TSS), and alternative last exon (transcription terminal site, TTS; Fig A in S1 File) were observed in our study. Of the 47,588 genes that were expressed during spermiogenesis, 91,112 instances of alternative splicing were detected at 19,127 genes (approximately 40.2% of the expressed transcriptome), indicating that each detected gene expressed one or more alternative splicing forms. In addition to 22,821 (25.0%) previously unidentified splicing sites, we confirmed 74.9% of known alternative splicing events.

We documented the occurrence of five common types of alternative splicing (Fig A in S1 File and Table B in S2 File). The two most abundant splicing events were TSS and TTS, which changes the transcription start site or terminal site. Exon skipping was only 10%, followed by IR (1.9%) and alternative exon ends (3.9%). Some transcript isoforms have not been identified previously, such as multiple skipped exons (0.86%), approximate boundary skipped exons splicing sites (0.9%), multiple retained introns (0.09%), approximate boundary splicing retained intron sites (0.49%). The alternative splicing junctions are listed in S1 Table.

**Fig 2:**
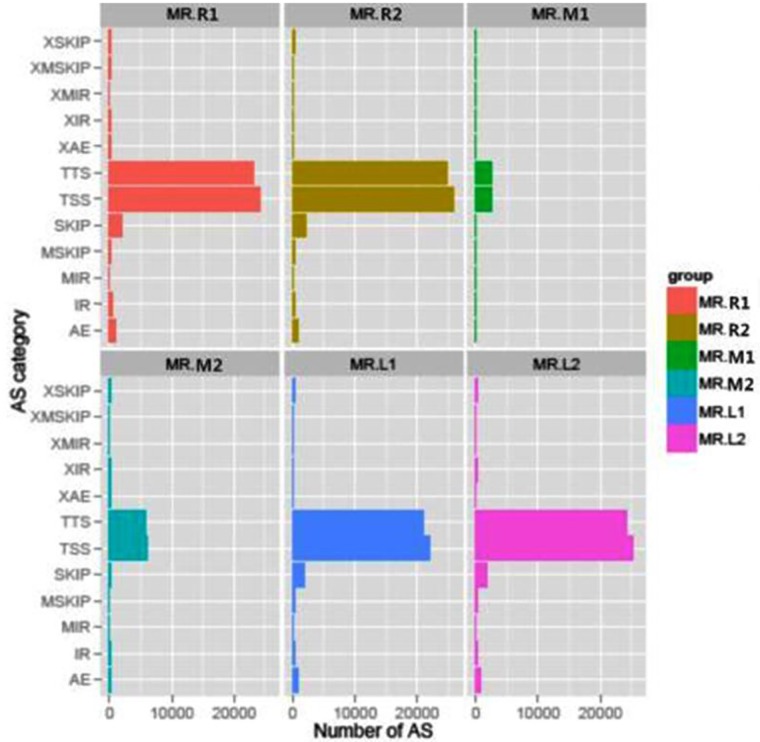
Number of common alternative splicing events in three types of cells during spermiogenesis.

To explore the role of alternative splicing events in the developmental regulation of gene expression, we examined the frequency of each class of alternative splicing across developmental zones. There was no substantial difference between the round and elongated spermatids, in which TSS and TTS were detected as the two major alternative splicing events, followed by SKIP and AE, and other types of alternative splicing events with very low percentages. However, the number of alternative splicing events, especially TTS and TSS, sharply decreased in sperm (Fig 2). These data suggest that alternative splicing events are likely to result in the different types and the functional variation of transcripts for each expressed gene in the mouse sperm.

### The expression trend of the differentially expressed genes in transcriptomic level during spermiogenesis

**Fig 3:**
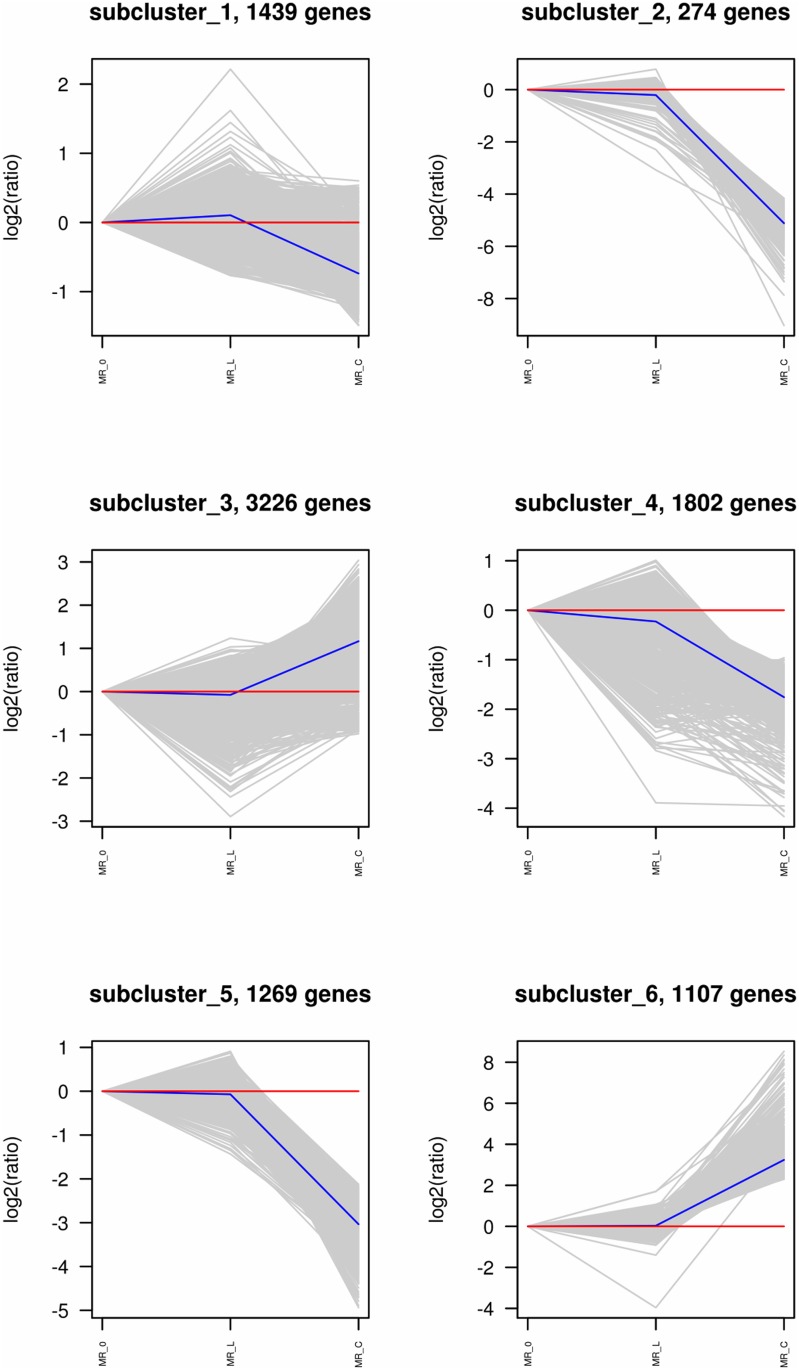
Complete K-means cluster maps based on the expression of differentially expressed genes (DEGs). Lines show the expression trends in each developmental stage (round spermatids, elongated spermatid, and sperm).

**Fig 4:**
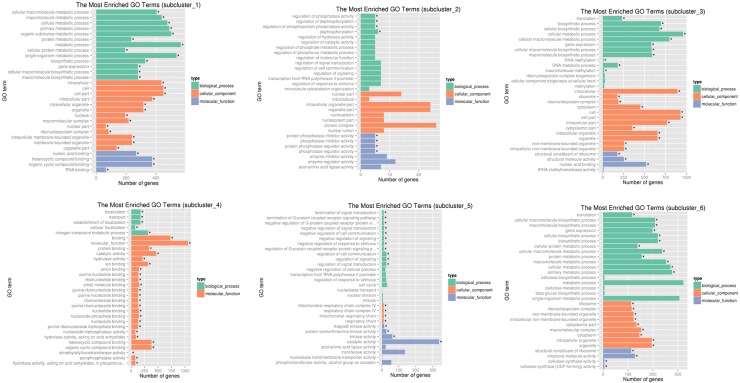
Functional enrichment results of each subcluster using NbClust R package. Asterisks (*) indicate the differentially expressed genes that were significantly enriched in GO terms.

Totally we identified 12,105 differentially expressed genes (DEGs). Among them 1,318 (538 upregulated and 780 downregulated) genes showed significant differential expression between the round and elongated spermatids. In the comparison of the two types of spermatids with sperms, 7,881 (3,679 upregulated and 4,202 downregulated) genes were differentially expressed in the elongated spermatids, and 10,899 (5,209 upregulated and 5,690 downregulated) genes significantly changed expression level in the round spermatids. These results demonstrated that fewer genes were differentially expressed between the two types of spermatids (round and elongated), compared with genes significantly changed expression level between the spermatids and sperms. Furthermore, more genes were down-regulated gradually with the progress in spermiogenesis. The genes were divided into 6 subclusters (Fig 3) based on their expression. Cluster 1 contained genes slightly upregulated when the round spermatids developed into elongated spermatids and downregulated when elongate spermatids matured into sperm. The expression of genes in clusters 2, 4, and 5 were gradually decreased at the elongated spermatid stage and sharply decreased in sperm, while the expression of genes in clusters 3 and 6 positively modulated in sperm after they showed steady expression in round and elongated spermatids. Around half of the DEGs (4,333) were upregulated among all the clustered DEGs (9,117) during development from elongate spermatids into sperm. We then used NbClust R package to assign genes to functional categories for each cluster (Fig 4).

### GO and KEGG pathway analysis results of DEGs

Gene distribution in enriched GO terms(Fig B-Fig D in S1 File) showed that most of the DEGs in the cell component (CC) term were enriched. The pathway enrichment results(Fig E-Fig G in S1 File) showed that some DEGs in the ribosome pathway were significantly enriched in round spermatids relative to elongated spermatids. The DEGs involved in the oxidative phosphorylation pathway, RNA transport pathway, ribosomes, spliceosomes, and protein processing in the endoplasmic reticulum were most significantly enriched in the elongated spermatids relative to those in sperms. A majority of DEGs in the transport pathway, oxidative phosphorylation, lysosome pathway, and mRNA surveillance pathway were significantly enriched in the round spermatids relative to those in sperm. We selected clear themes, particularly those based on the physical and biochemical changes during mouse spermiogenesis, and further divided them into 15 groups based on function (Table C in S2 File). One of the significant changes during spermiogenesis is the gradual formation of acrosome from the Golgi apparatus, and 119 and 17 genes were enriched in Golgi apparatus and acrosomes, respectively. The morphology, distribution, and function of mitochondria in haploid germ cells were changed after meiosis, and 118 genes were found enriched in mitochondria in our data. A total of 76, 57, 13, and 17 genes were responsible for chromosome condensation, histone degradation, protamine synthesis, and ankyrin, indicating that histones associated with chromatin are replaced by protamines during the process of spermiogenesis, and eventually chromatin is remodeled. A total of 71 microtubule and 19 flagellum genes were enriched, suggesting that motion transition occurs during spermiogenesis. Furthermore, 41, 19, 23, and 15 genes related to transcription factors, translation initiation factor activity, spliceosomes, and ncRNA metabolic process, respectively, were enriched, suggesting that transcription and splicing, translation, and ncRNA metabolic process have considerable influence in spermiogenesis. Then, 13 and 11 genes related to centrioles and the MAPK signaling pathway, respectively, were enriched, indicating that centrioles and signaling regulation also vary during the process of spermiogenesis. The enriched DEGs in these themes are characterized in detail in S2 Table.

### The expression trend of the transcription factors during spermiogenesis

We detected 41 DEGs related to the transcription process during spermiogenesis, with several transcription factors showing significantly higher expression in round and elongated spermatids in this study. However, all of them were obviously downregulated in sperm (S2 Table). The variations of the transcriptional factors at the different stages of spermiogenesis suggested that transcriptional regulation of gene expression underlies the major events in spermiogenesis. The decreases of these transcription factors indicate that the transcription activity is gradually lowered during spermiogenesis.

### Real-time RT -PCR Analysis

**Fig 5:**
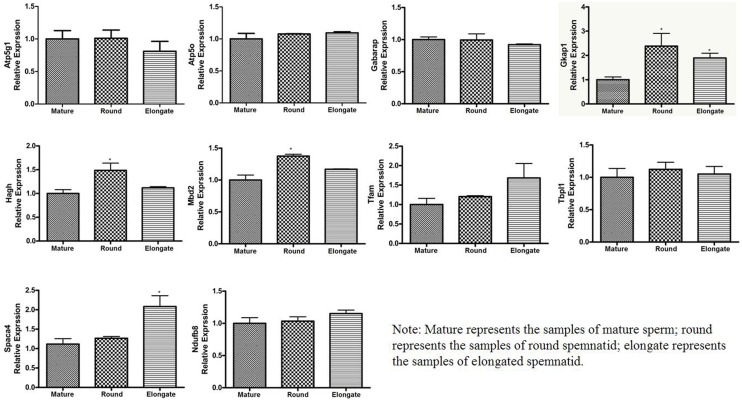
Relative levels of expression of ten genes in mature sperm, round spemnatid and elongated spemnatid of Mus musculus. The transcript levels in each sample were normalized to that of the β-actin (internal control). Gene expression data were obtained using the 2^-ΔΔCt^ relative quantitative method. Student’s t-test was used for statistical analysis (*P < 0.05).

The expression of ten randomly selected DEGs, playing some kind of roles in the physical and biochemical changes during spermiogenesis, was measured using quantitative real-time PCR (qPCR), and the expression quantities were normalized to β-actin (Fig 5). The expression level of these 10 transcripts were higher in round and elongated spermatids than in sperm, which was consistent with the variations from RNA-seq analysis. Among them, eight transcripts were significantly different during spermiogenesis (*P<0*.*5*) while the other two were not (*P>0*.*5*).

## Discussion

We performed deep transcriptomic surveys of three types of male haploid mouse cells during spermiogenesis, and mapped the transcriptional changes in the development process in detail. Our results revealed extensively transcriptional activity in the process of spermiogenesis, and these findings are consistent with the expression trends of protamine and transition protein in postmeiotic germ cells revealed in a previous study [3].

With the help of the LCM technology, we successfully captured the target cell samples of the round spermatids and elongated spermatids. Although spermatogenic lineage development is a complex process, all types of the germ line cells locate in a spatio-temporal manner in the spermatogenic cycle. The order of germ line cells residing from the luminal side to the basement membrane of the seminiferous tubules is: elongated spermatid, round spermatid, secondary sprmatocyte, primary spermatocyte and spermatogonia. The elongated spermatids reside separately on the closest layer of the luminal side of seminiferous tubules, while the round spermatids are right next to the elongated spermatids. In addition, the shapes of these two types of cells are obviously distinct. The elongated spermatids are slender, while the round spermatids are circle or round[14–16]. Hence, the homogeneity of the two types of cells analyzed in this study is highly reliable.

The spermatid transcriptome varied during spermiogenesis. Progress in the spermatid development resulted in a decrease in the total reads, uniq-mapped reads, and map rate. This trend is consistent with the trend of variation of transcription during spermiogenesis. Compared with somatic cells, more reads (up to 35%) were mapped to introns in sperm since some introns might be spliced as exons that have not yet been added to the gene splicing models, which indicated that the splicing mechanism in sperm varied from that in spermatids and somatic cells. A previous study showed that substantially more genic and intergenic regions were transcribed in spermatids than in other somatic cells [7]. Our report of up to 52% of the intergenic reads in sperm suggested that pseudogenes and transposable elements might be plentiful in sperm and ncRNA may play a very important role in regulating the expression of genes in sperm.

The alternative splicing types varied during spermiogenesis. Our dataset showed 14.4–16.7% of splice-spanning reads in round and elongated spermatids, and 0.2–0.5% in sperms (Table A in S2 File), and approximately 3% in somatic cells [9]. The results of the mapping indicated that the splicing rate was high in round and elongated spermatids, which is in agreement with a previous report [7], but the splicing rate in sperm in our study was much lower. The splicing instance in spermatids differed considerably than that in somatic cells, and TSS and TTS were the most frequently encountered events, suggesting that these events play important roles in spermiogenesis [17]. Our results suggested that transcription levels were lowered and splicing events were reduced with the progression of spermiogenesis.

Different genes showed different levels of transcription during spermiogenesis. We detected 12,105 DEGs and 5,104 de novo genes (S3 Table). The remaining predicted genes that were not detected in the mouse are likely to be exclusively required for sperm development. Haploid round spermatids undergo complex morphological, biochemical, and physiological modifications, resulting in the formation of spermatozoa. To ensure efficient transmission of the paternal genome from the spermatozoa to the oocyte at fertilization, the composition and compaction of chromatin undergo profound changes in spermatids, resulting in a species-specific shape of sperm head, acrosome development, and flagellum formation. Consistently, a large number of genes related to these traits were differentially expressed in our study. Throughout this process, even though the total transcript number was gradually decreased in our study, nearly half of the DEGs were upregulated from round spermatids to sperm (5,209 upregulated and 5,690 downregulated). In particular, we found an increase in the abundance of transcripts for transition proteins and protamines, the principal basic nuclear proteins in the round and elongated spermatids in our study. At the same time, genes involved in helicase activity, histone deacetylase, dynein complex synthesis, protamine synthesis, acrosomal matrix synthesis, and mitochondrial ribosome synthesis were upregulated. Especially, the upregulation of transition proteins and protamines in round spermatids and elongated spermatids were also reported in previous studies [18–20]. The results indicated that stringent temporal and stage-specific gene expression was a prerequisite for the correct differentiation of round spermatids into spermatozoa.

The expression of transcription factors varied during spermiogenesis. Previous studies have uncovered a few potential regulators of mouse spermatid development. Transcription is regulated via methylation [7] and trans-acting factors that bind to the TATA-box [21] or other specific DNA sequences in the promoter region [7], and the transcription activity in the spermatid was reported to gradually stop. The transcripts are protected and stored as ribonucleoprotein particles in a translationally repressed state for several days or longer and are translated in elongated spermatids or in the zygote [6,22,23]. In our study, nearly half of the DEGs were upregulated during spermiogenesis, which indicated that the transcription activity in spermatids was downregulated but might not completely stop. Furthermore, approximately 41 transcription factors were differentially expressed in mouse spermiogenesis, of which 11 were significantly upregulated while others were significantly downregulated, and these transcription factors may help maintain the transcription activity in spermiogenesis. These genes are excellent candidates for future functional genomics studies to explore the transcription activity in spermatids.

Initiation of transcription in mammalian mitochondria depends on three proteins: mitochondrial RNA polymerase (*POLRMT*), mitochondrial transcription factor A (*TFAM*), and mitochondrial transcription factor B2 (*TFB2M*). *TFAM* is an important regulator in maintaining the mitochondrial DNA copy number, which is necessary to organize mitochondrial chromatin and in nucleoid formation [24]. *TFAM* overexpression can reduce mitochondrial permeability transition and ameliorate delayed neuronal death in the hippocampus after transient forebrain ischemia [25]. Perhaps the high *TFAM* expression in mice suggests that transcription in mouse spermiogenesis may play an important role in maintaining the stability of mitochondria.

The onset of TATA-binding protein *(TBP)* overexpression is associated with the appearance of the first haploid cells. The *TBP* mRNA levels in these cells were more than 1000-fold greater than those in somatic cell types [21]. *TBP* was overexpressed in the spermatids in our study, and TBP-associated factors such as *Taf9* and TBP-like 1 (*Tbpl1*) were also very abundant. As *TBP* plays an important role in transcription regulation, and there is about 10% sites containing histones in nucleosome in mouse spermatozoa [26,27], the high expression of cell type-specific genes may indicate that the gene expression during spermiogenesis is different from somatic cells.

We detected high expression levels of polymerase (RNA) II (DNA-directed) polypeptide I (*Polr2i*) in spermatids in our study. *Polr2i* participates in the transcription of DNA into RNA (http://www.uniprot.org/uniprot/P60898), and in the purine and pyrimidine metabolism pathways, mRNA splicing, and estrogen signaling. Ubiquitin-specific peptidase 16 (*Usp16*) was also highly expressed in both round and elongated spermatids in our study. *Usp16* positively regulates DNA-dependent transcription and plays a role in histone deubiquitination and chromatin modification. Homeodomain-interacting protein kinase 1 (*Hipk1*) is important in transducing growth-regulatory signals and modulating the localization and phosphorylation of the Fas death domain-associated protein (Daxx) [28], and it was highly expressed in spermatids. The present study indicated that *Hipk1* not only mediated cell proliferation and apoptosis in response to morphogenetic and genotoxic signals during mouse development [29] but also promoted the development of spermiogenesis.

The genes for transition proteins and protamines were abundantly transcribed in round and elongated spermatids in our study, such as ubiquitin-conjugating enzyme E2B (*Ube2b*), sirtuin 2 (*Sirt2*), ornithine decarboxylase structural 1 (*Odc1*), and glutamate-ammonia ligase (*Glul*). *Ube2b*-deficient mice showed male infertility and sperm head shape anomalies [30]. *Sirt2* takes part in histone acetyltransferase binding. Glul plays an important role in arginine and proline metabolism, and *Odc1* participates in the arginine and proline metabolism, glutathione metabolism, and polyamine biosynthesis pathways. The genes for chromatin modification, including ubiquitin-specific peptidase 16 (*Usp 16*) and *Ube2b*, were overexpressed in our study. These genes play an important role in maintaining chromatin states that are transcriptionally permissive, and are presumably associated with the continuous repackaging of DNA during spermiogenesis. Our results are in agreement with previous observations that substantial remodeling of chromatin during spermiogenesis involves the replacement of standard histones [7,31].

The transcript number and alternative splicing events gradually decreased during spermiogenesis. However, the alternative splicing events, DEGs, and the transcription trends differed. Compared with round and elongated spermatids, the splicing rate was lower and more reads were mapped to intergenics and introns in sperm, and the TTS and TSS splicing events were sharply decreased in sperm. The up-regulation of nearly half of the DEGs may be not only related to organelle formation and function but also to histone degradation, protamine synthesis, chromosome condensation, and transcription. This variation in the transcript level during spermiogenesis indicated that the transcription activity was gradually lowered but might not terminate in sperm.

## Supporting Information

S1 File The figures of alternative splicing events, gene distribution in enriched GO terms and pathway enrichment results. (PDF)

S2 File The tables of Summary of read number, Common alternative splicing events, fifteen functional categories of DEGs and primer sequences of qPCR. (PDF)

S1 Table The alternative splicing events. (XLS)

S2 Table The GO and KEGG pathway analysis results of DEGs. (XLS)

S3 Table De novo genes. (XLS)
