# Supplementary material for: Transcriptomic Variation during Spermiogenesis in Mouse Germ Cells
Source: PLoS One. 2016 Nov 11;11(11):e0164874. doi: 10.1371/journal.pone.0164874 (PMC5105947; doi:10.1371/journal.pone.0164874)

Figure A

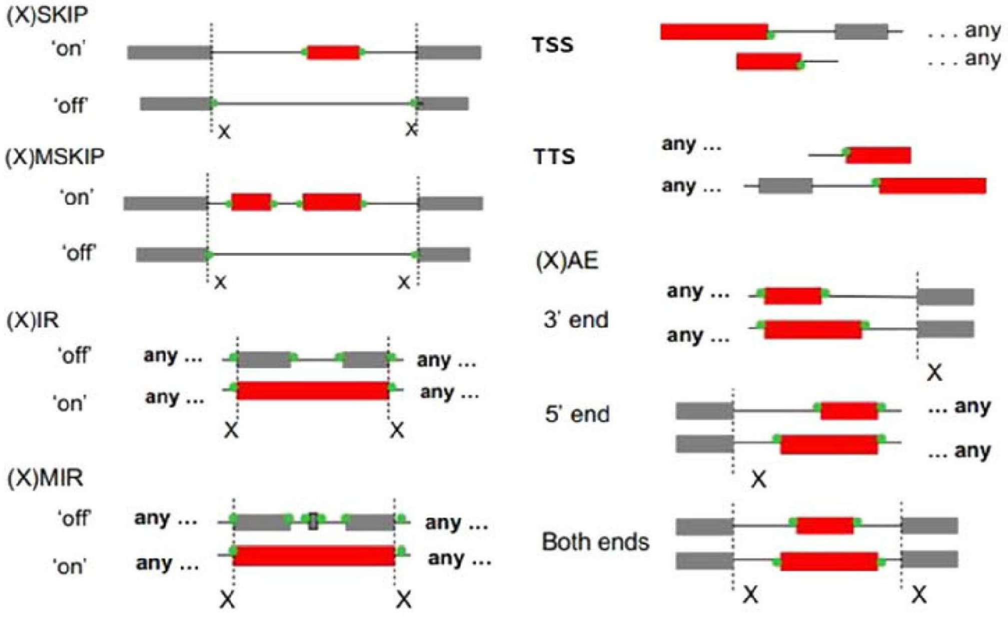


Figure B (MR_LvsMR_M)

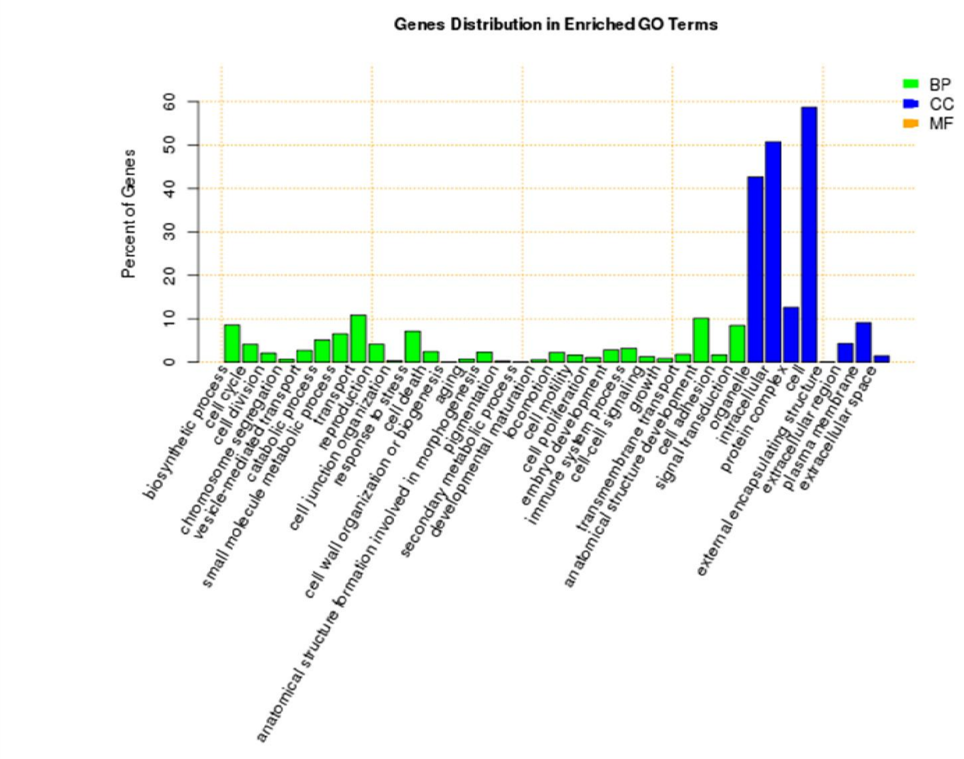

Figure C (MR_RvsMR_L)

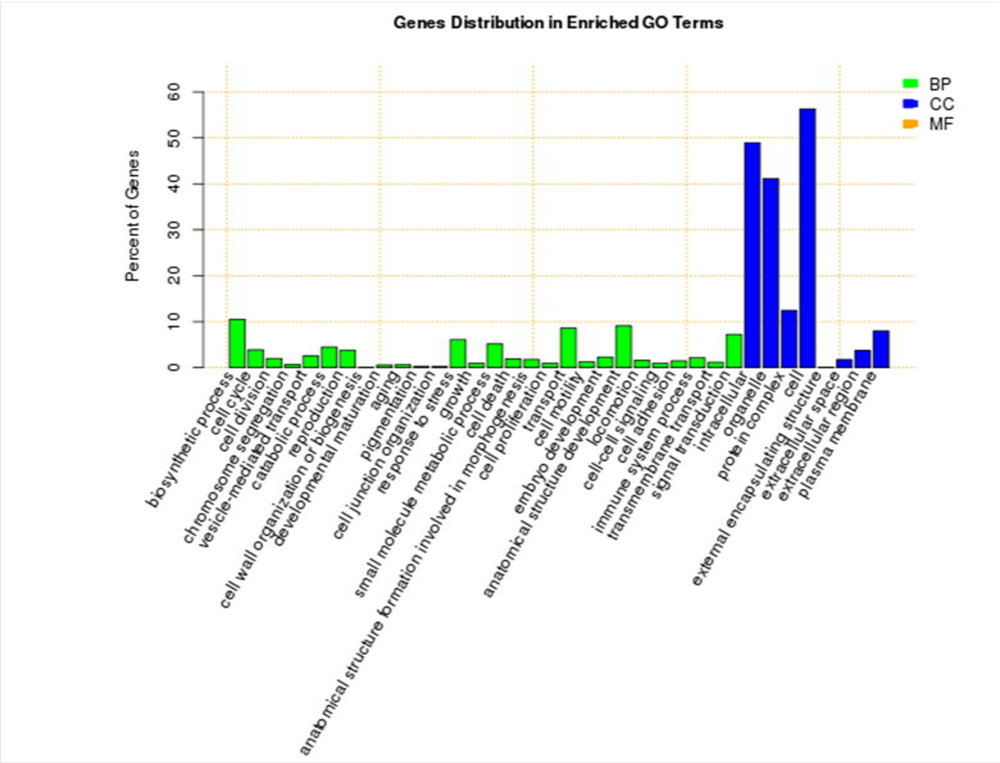


Figure D (MR_RvsMR_M)

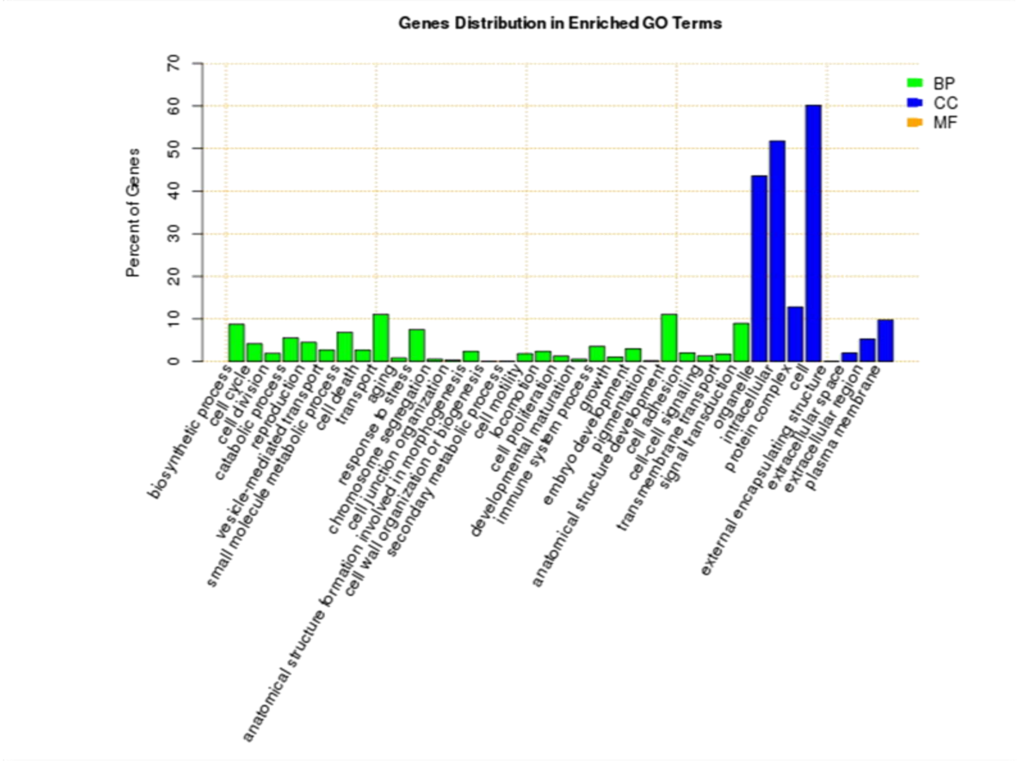


Figure E (MR_RvsMR_M.Pathscatter)

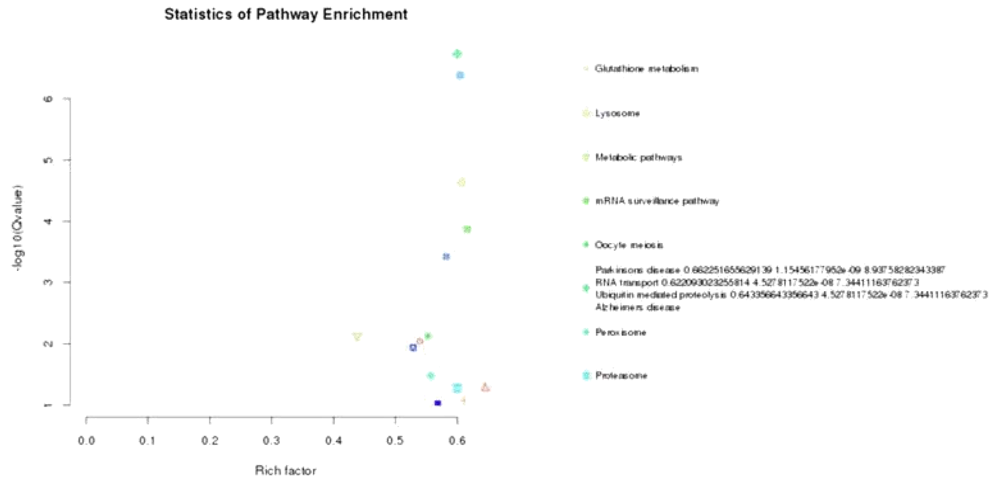


Figure F (MR_LvsMR_M.Pathscatter)

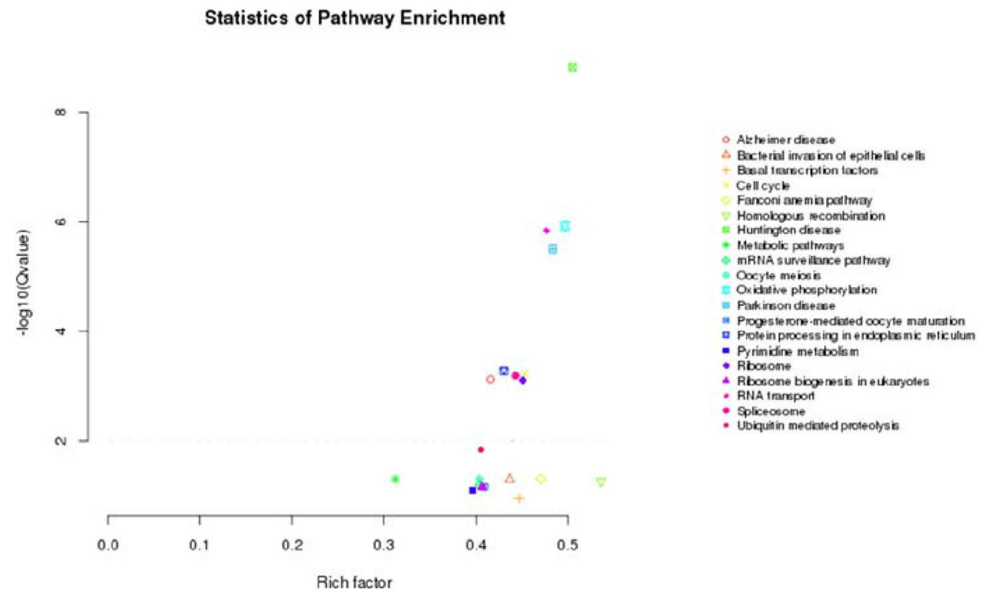

Figure G (MR_RvsMR_L.Pathscatter)

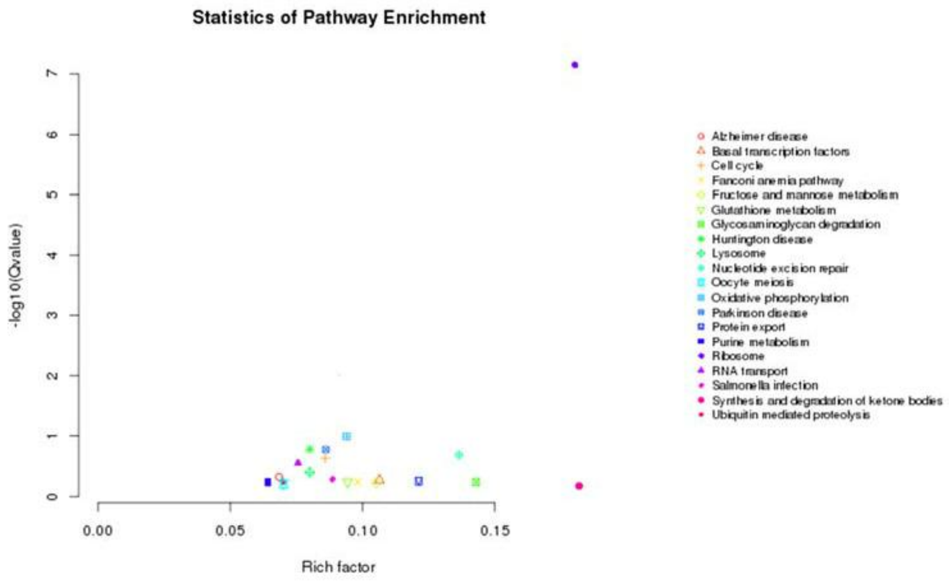

Supplement: S1 File — (PDF) [file pone.0164874.s001.pdf]
