# Supplementary material for: Transcriptomic Variation during Spermiogenesis in Mouse Germ Cells
Source: PLoS One. 2016 Nov 11;11(11):e0164874. doi: 10.1371/journal.pone.0164874 (PMC5105947; doi:10.1371/journal.pone.0164874)
Supplement: S2 File — (PDF) [file pone.0164874.s002.pdf]

Table A Summary of read number

Mapping Summary	R1	R2	L1	L2	M1	M2
Total Reads	81823434	97229756	56366502	95095060	47101384	49566142
Total Mapped Reads	69433435	83783153	48600531	81573996	33190081	34540993
Uniq-Mapped Reads	63971761	81782329	47417172	79577620	31726185	33063076
Muilti- Mapped Reads	1848499	2000824	11833859	1996367	1463896	1463896
Read-1	32512972	41181824	23814353	39774016	15867742	16513211
Read-2	31458789	40600505	23602819	38903604	15858443	16513211
Reads map to '＋'	31940565	40823334	23666171	39741296	15846826	16536620
Reads map to '—'	32031196	40958995	23751001	39836324	15879359	16526456
Non-splice reads	52162994	65545223	38343370	64320295	31620334	32822369
Splice reads	11808767	16237106	9073802	15257325	105851	240707
Splice rate	18.50%	19.85%	19.14%	19.17%	0.33%	0.73%
Map_ Rate	84.86%	86.17%	86.22%	85.78%	70.47%	69.69%

Table B Common alternative splicing events in short and elongated spermatids and sperm during spermiogenesis

**Alternative transcript events**	**Number detected (R1)**	**Number detected (R2)**	**Number detecte d (L1)**	**Number detected (L2)**	**Number detected (M1)**	**Number detected (M2)**
**Exon skipping**	**2298**	**2606**	**2180**	**2216**	**6**	**122**
SKIP	1886	2126	1760	1822	4	102
XSKIP	166	226	222	190	0	14
MSKIP	208	218	176	174	0	6
XMSKIP	38	36	22	30	2	0

**Intron retention**	**380**	**444**	**344**	**376**	**44**	**114**
IR	278	328	246	286	20	60
XIR	92	106	92	80	22	52
MIR	6	6	6	4	0	2
XMIR	4	4	0	6	2	0
**Alternative exon ends (5', 3', or both)**	**882**	**927**	**795**	**830**	**15**	**89**
AE	818	871	757	778	12	76
XAE	64	56	38	52	3	13
**Alternative first exon**	**24208**	**25935**	**21881**	**25257**	**2518**	**6006**
**Alternative last exon**	**23027**	**24733**	**20870**	**24299**	**2491**	**5846**
**Total**	50795	54645	46070	52978	5074	12177

Table C Fifteen functional categories of DEGs in mouse spermatids during spermiogenesis

Functional Category	Terms Included	Number of Genes
Golgi apparatus	GO:0005794 GO:0044431 GO:0005802 GO:0048193 GO:0006888 GO:0005802 GO:0048193 GO:0005798	119
Acrosomal	GO:0043159 GO:0001669 GO:0001669	17
Centriole	GO:0005814	13
Mitochondrion	GO:0005739 GO:0005761 GO:0005759	118
Microtubule	GO:0007017 GO:0000226 GO:0044450	71

Flagellum	GO:0005930 GO:0019861	19
histone degradation	GO:0004386 GO:0042826 GO:0043967 GO:0016570	57
Protamine synthetizing	mmu00330	13
Ankyrin	IPR002110	17
chromosome condensing	GO:0006461 GO:0000794 GO:0000793 GO:0016585 IPR000408	76
transcription factor	GO:0006351 GO:0008134 mmu03022 GO:0030528	41
translation initiation factor activity	GO:0003743	19
MAPK signaling pathway	mmu04010	11
ncRNA metabolic process	GO:0034660	15
Spliceosome	mmu03040	23

Table D Primer sequences used for qPCR and relative levels of expression of ten genes in mature sperm, round spemnatid and elongated spemnatid of Mus musculus

Gene Name	**Primers Sequence (5'-3')**	**Size (bp)**
Gkap1	Forward: TGGGAAACAAAGCAACCT<br>Reverse: CAAATGGAATGGGAGGAT	136
Gabarap	Forward: ACAATGTCATTCCACCCA<br>Reverse: ACAGACCATAGACGCTTTC	111
Tbpl1	Forward: TCATGCCAGCTATGAACC<br>Reverse: CAGTCGCCACAGCCTTTA	131
Spaca4	Forward: GCCTCACATACAGTCTCACCA<br>Reverse: TGAAGCAGCAGCAACAGG	130
Ndufb8	Forward: TTCGGCTTTGTGGCTTTC<br>Reverse: CGCTCCAGGTACAGATTATTG	110
Atp5g1	Forward: ATGCCAGGAACCCATCTC<br>Reverse: GGAAGGCGACCATCAAAC	102

Mbd2	Forward: GGAGTCGGTCCAGGTAGCA<br>Reverse: AAGCCAAACAGCAGGGTTC	126
Hagh	Forward: GAAGGTTTATGGAGGTGATG<br>Reverse: AGTATGGCAGGGTGTTGA	115
Atp5o	Forward: TTCAGGTCTACGGCATCG<br>Reverse: GCAGCAGCTCCTTCTCCA	96
Tfam	Forward: GGAATGTGGAGCGTGCTA<br>Reverse: GCTACCCATGCTGGAAAA	126
β-actin	Forward: ACTCGCTGCGCTCGGTCGTT<br>Reverse:CCTTTTGCTGGCCTTTTGCTCAC	125
